# Short lifespan of syngeneic transplanted MSC is a consequence of in vivo apoptosis and immune cell recruitment in mice

**DOI:** 10.1038/s41419-021-03839-w

**Published:** 2021-06-02

**Authors:** Mihai Bogdan Preda, Carmen Alexandra Neculachi, Ioana Madalina Fenyo, Ana-Maria Vacaru, Mihai Alin Publik, Maya Simionescu, Alexandrina Burlacu

**Affiliations:** 1 Laboratory of Stem Cell Biology, Institute of Cellular Biology and Pathology “Nicolae Simionescu”, Bucharest, Romania; 2 Laboratory of Gene Regulation and Molecular Therapies, Institute of Cellular Biology and Pathology “Nicolae Simionescu”, Bucharest, Romania

**Keywords:** Apoptosis, Mesenchymal stem cells

## Abstract

Mesenchymal stromal cells (MSC) are attractive tools for cell-based therapy, yet the mechanisms underlying their migration and survival post-transplantation are unclear. Accumulating evidence indicates that MSC apoptosis modulates both innate and adaptive immune responses which impact on MSC therapeutic effects. Using a dual tracking system, namely the Luciferase expression and VivoTrack680 labelling, and in vivo optical imaging, we investigated the survival and migration of MSC transplanted by various routes (intravenous, subcutaneous, intrapancreatic and intrasplenic) in order to identify the best delivery approach that provides an accumulation of therapeutic cells to the injured pancreas in the non-obese diabetic (NOD) mouse. The results showed that transplanted MSC had limited migration capacity, irrespective of the administration route, and were short-lived with almost total disappearance at 7 days after transplantation. Within one day after transplantation, cells activated hypoxia signalling pathways, followed by Caspase 3-mediated apoptosis. These were subsequently followed by local recruitment of immune cells at the transplantation site, and the engulfment of apoptotic MSC by macrophages. Our results argue for a “hit and die” mechanism of transplanted MSC. Further investigations will elucidate the molecular crosstalk between the inoculated and the host-immune cells.

## Introduction

Mesenchymal stromal cells (MSC) have potent immunomodulatory properties, making these cells rational candidates for the treatment of autoimmune diabetes and other immune disorders^1,2^. However, the implementation of MSC-based therapies still imposes numerous considerations that impact the clinical outcome, including the route of administration, the fate of cells after transplantation, the pharmacokinetics and biological properties of the transplanted MSC^3^.

Conflicting data have been reported in reference to MSC survival after transplantation in mice^4–7^. Nevertheless, accumulating evidence indicates that in vivo apoptosis affects the transplanted cells and this process could impact the mechanism of MSC-mediated immunosuppression. The “dying stem cell hypothesis”, by which the apoptosis of transplanted MSC modulates the innate and adaptive immune responses, was first formulated in 2005 (ref. ^8^), and later on experimentally reinforced under variable pathological conditions by several groups^9–12^.

The murine models of inflammatory insulitis, such as the non-obese diabetic (NOD) mouse, have been extensively used as preclinical models for studying autoimmune diabetes and testing immunotherapies^5,13–15^. Several studies suggested that local or systemic administration of MSC prevented the onset of diabetes and reversed hyperglycaemia in diabetic NOD mice^4,16–18^. However, the mechanisms underlying the process of MSC recruitment and migration to target sites, as well as the distribution and fate of MSC when administered by various routes remain elusive. Several approaches to improve MSC therapeutic efficacy have been proposed, such as pre-transplantation priming with hypoxia, in vitro treatment with small molecules or cytokines, or encapsulation with biomaterials, which all mainly triggered improved cell retention and survival post-transplantation^3,19,20^.

In this paper, we explored the distribution pattern and the viability of syngeneic MSC adoptively transferred in NOD mice in order to identify the optimal route of administration that provides prolonged survival and/or targeted migration of cells to the pancreatic islets. By employing different administration routes, i.e., intravenous, intrapancreatic, intrasplenic and subcutaneous, and in vivo optical imaging for body-wide detection of two reporter molecules, namely Luciferase (Luc) and VivoTrackT680 (VT680) fluorescent dye, we provide evidence that shortly after transplantation, MSC become hypoxic, activate Caspase 3-mediated apoptosis and are engulfed by locally accumulating macrophages.

## Results

### MSC survival and migration in NOD mice

Therapeutic attempts for diabetes using MSC raised the question about the potential of these cells to migrate to the inflamed pancreatic islets. As intravenous administration is currently the most popular route of therapeutic MSC delivery^21^, we first studied the distribution of intravenously injected syngeneic MSC in pre-diabetic NOD females. MSC harvested from the bone marrow of male NOD mice were grown in culture for six passages and then lentiviral transduced to express Luc. Soon after intravenous administration, bioluminescence imaging (BLI) showed a localized signal in the lungs, which gradually decreased within the next days, until undetectable at 7 days post-transplant. However, a low specific BLI signal still persisted in the lungs, as proven by ex vivo organ imaging at 7 days post-cell infusion, and no signal was detected in other organs (Fig. S1A–C). These results indicated that the intravenous route was not effective to target MSC to the pancreas.

**Fig. 1:**
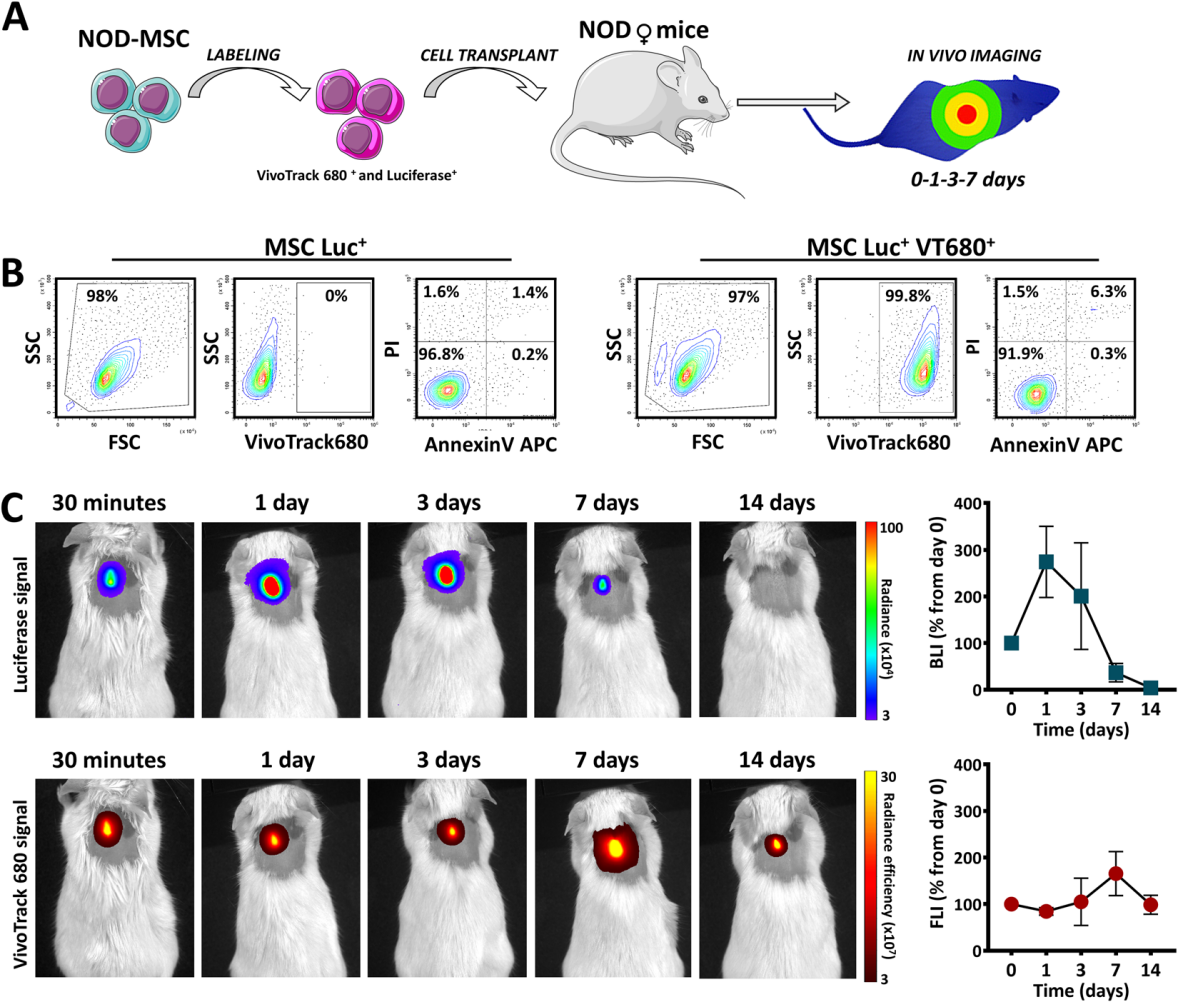
Survival of MSC after in vitro double labelling with Luciferase and VT680 followed by subcutaneous transplantation into NOD female mice. **A** Schematic representation of the experimental workflow. This figure was made with Servier Medical Art templates, which is licensed under a Creative Commons Attribution 3.0 Unported License: https://smart.servier.com. **B** Preservation of cell viability after in vitro labelling of Luc^+^MSC with VT680. Note than VT680 labelling produced minimal changes in cell viability of Luc^+^MSC and that the majority of cells were not affected by the second labelling. **C** The time-course images of BLI and FLI signals produced by double-labelled MSC after subcutaneous transplantation in a NOD mouse. The quantitative data from five mice is given on the right side. Data are plotted as mean ± SEM. Note the high increase in the BLI signal at day 1. However, due to the large variability in the animal responses, the increase is not statistically significant (one-way ANOVA followed by Tukey’s test).

We next evaluated the in vivo distribution and survival of MSC following the subcutaneous transplantation, which was previously reported as a route that provided prolonged survival of the transplanted cells^22^. To this aim, MSC were double-labelled with Luc and VT680 and 10^6^ cells were subcutaneously grafted in NOD mice (Fig. 1A). Before transplantation, the preservation of cell viability after VT680 incorporation into Luc+ cells was certified by flow cytometry, after staining with Annexin V and Propidium Iodide (Fig. 1B). The in vivo kinetics of double-labelled MSC was assessed by comparative multimodal BLI and fluorescence imaging (FLI) for Luc and VT680 signals, respectively. The results showed that FLI signal persisted at similar intensities for at least 14 days. However, the BLI signal did not paralleled the FLI signal and showed a significant reduction in intensity at 7 days post-transplant, with complete clearance at 14 days. To check whether the anatomical region influenced the fate of the transplanted cells, the survival of Luc^+^ MSC was comparatively evaluated in mice with subcutaneous cell transplants in the interscapular and inguinal regions. The results showed similar BLI patterns of the two anatomical regions, after subcutaneous transplantation of Luc^+^ MSC (Fig. S1D–E), suggesting that the limited survival of syngeneic MSC after transplantation in the NOD prediabetic mouse did not depend on the anatomical region of subcutaneous cell injection.

**Fig. 2:**
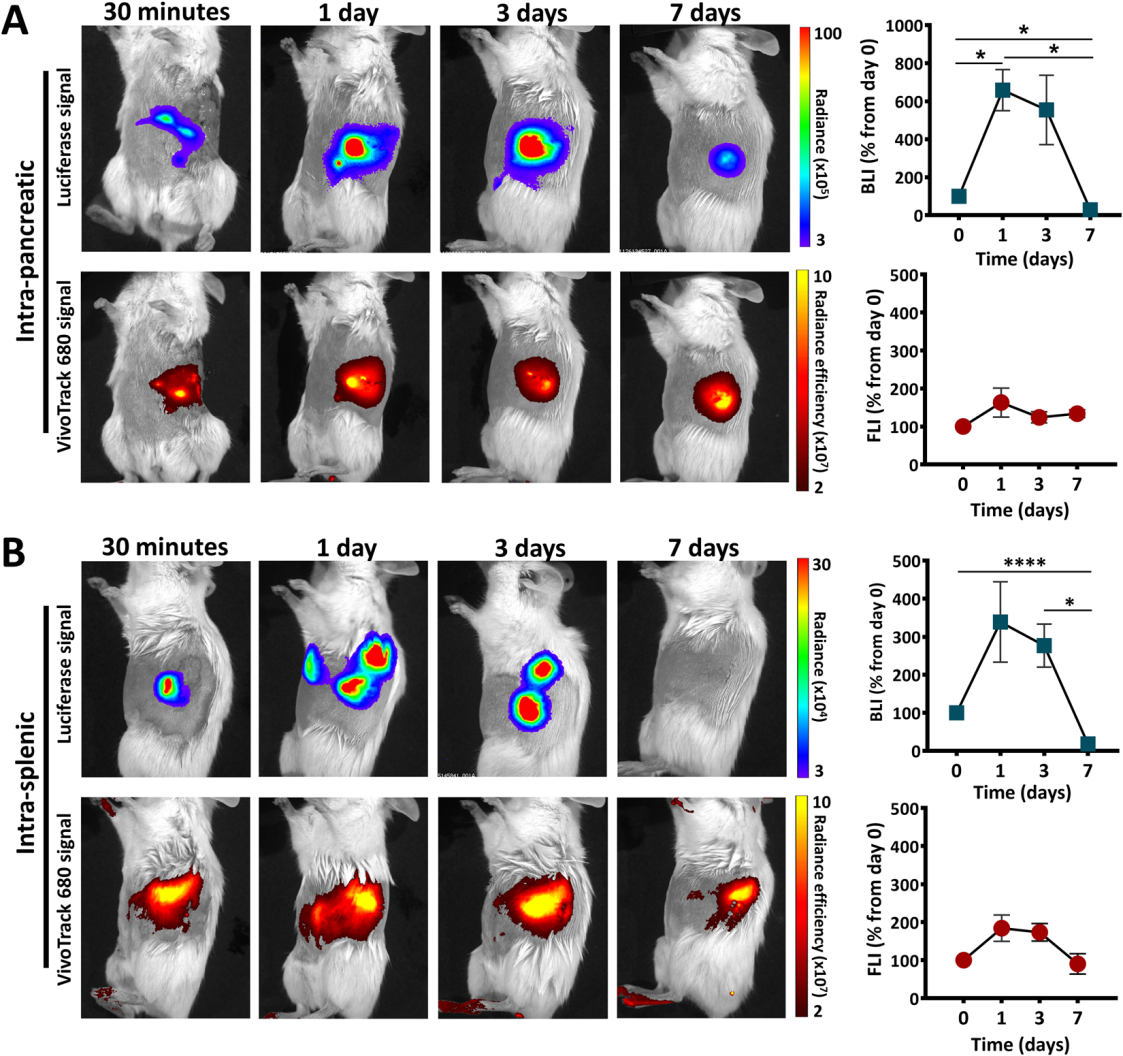
Biodistribution of MSC after intrapancreatic or intrasplenic transplantation in pre-diabetic NOD females. BLI and FLI time-course images of a representative mouse transplanted with Luc^+^ VT680^+^ MSC into the pancreas (**A**) and spleen (**B**). The diagrams on the right illustrate the quantitative data from all mice within each group (*n* = 5–6 per group). Data are plotted as mean ± SEM (*p < 0.05, one-way ANOVA followed by Tukey’s test).

Next, alternative routes have been evaluated, i.e., the intrapancreatic and intrasplenic transplantation, aiming at increasing the survival time and the accumulation of transplanted cells to the pancreas. For these experiments, 5 × 10^5^ double-labelled MSC were directly grafted either into the pancreas or the spleen of pre-diabetic NOD females, following the same protocol described above (Fig. 1A). The results showed that FLI signal persisted at similar intensities for at least 7 days for all the mice within both transplanted groups. However, the BLI signal intensity showed a significant reduction at 7 days post-transplant, when only three out of five animals within the intrapancreatic group and only one out of six animals within the intrasplenic group, displayed a small BLI signal (Fig. 2A, B). A possible interpretation of these data is that MSC died within days after the transplant (abrupt decline or loss of Luc signal), while the fluorescent signal persisted in the tissue, giving a false-positive response regarding the location of the transplanted cells.

Interestingly, a significant increase of the BLI signal was detected in both groups at days 1 and 3 post-transplantation, which apparently might be an indicative of cell proliferation in vivo (Fig. 2A, B). However, this was followed by a rapid loss of BLI signal, thus the transient cell proliferation was ranked as very unlikely and consequently this issue was further addressed in the next studies.

### The microenvironment impacts the fate of the transplanted cells

**Fig. 3:**
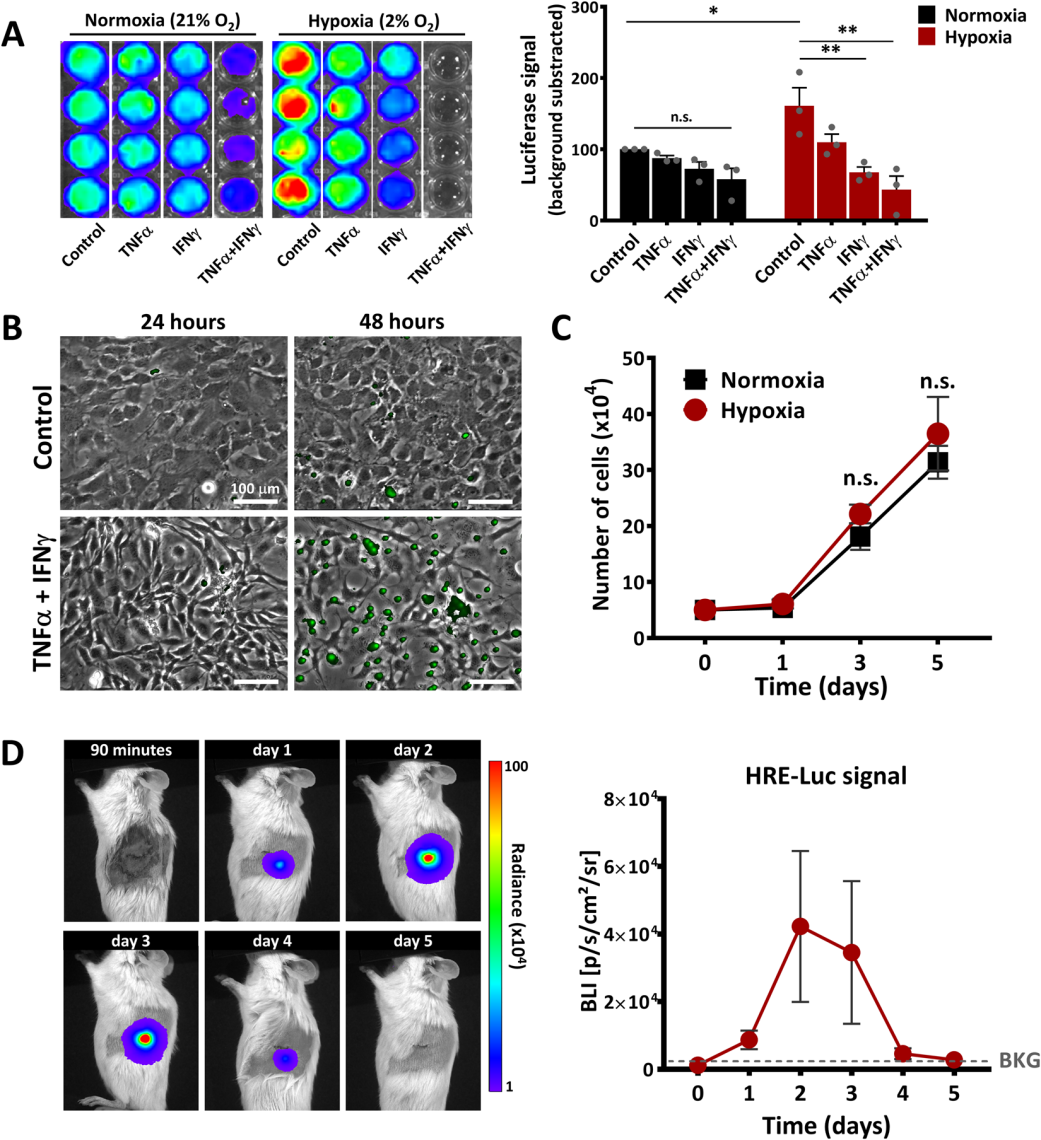
The behaviour of MSC in hypoxic environment. **A** The increase in Luc expression under the PGK promoter in Luc^+^ MSC, following in vitro exposure for 24 h to hypoxia and/or the pro-inflammatory cytokines TNFα and IFNγ. Data are plotted as mean ± SEM from four biological replicates (*p < 0.05, two-way ANOVA followed by Tukey’s test). **B** Phase-contrast microscopy illustrating the apoptosis activation in MSC cultured under hypoxic conditions (2%O_2_) in the absence (Control) or presence of inflammatory cytokines (TNFα + IFNγ). Apoptosis was detected by time-lapse fluorescence microscopy using CellEvent™ Caspase-3/7 reagent. Note that the cells are not affected by hypoxia per se, yet the presence of pro-inflammatory cytokines induced a massive rate of apoptosis after 48 h in vitro. **C** The proliferation curve of MSC under normoxia and hypoxia conditions within a 5-day interval. Note the minor and not-significant increase in MSC number under hypoxic conditions, as compared to normoxic conditions. **D** The time-course images of BLI signal produced by HRE-Luc-MSC after intra-pancreatic transplantation in a NOD mouse. The BLI signal denotes the local activation of hypoxia. The quantitative data from five mice is given on the right side. Data are plotted as mean ± SEM. Note the high increase in the BLI signal at days 2 and 3. However, due to the large variability in the animal responses, the increases were not statistically significant (one-way ANOVA followed by Tukey’s test).

Hypoxia and inflammation were reported to play important roles in modulating the properties of the transplanted cells^23^. Since the Luc expression used in our experimental model was driven by the promoter of phosphoglycerate kinase (Pgk), previously reported to be activated by low oxygen concentrations^24^, next we evaluated whether the Luc signal was affected by hypoxia and/or inflammatory conditions. To this aim, MSC were exposed in culture to mild hypoxia conditions (2% O_2_) and/or to the proinflammatory cytokines TNFα and IFNγ. The results showed that 24-h hypoxia resulted in increased BLI signal, as compared to normoxia (Fig. 3A). This data suggested that the increase in BLI signal was the consequence of the hypoxia-induced Pgk promoter activation. However, a decrease of Luc signal was measured in the presence of TNFα and IFNγ, thus suggesting that Luc signal might be altered by microenvironmental conditions. As IFNγ in the presence of TNFα has been previously reported to induce MSC apoptosis^25^, the impact of hypoxia on the incidence of this process was first assessed in vitro by time-lapse microscopy. In order to evaluate apoptosis, CellEvent™ Caspase-3/7 reagent was added in the culture medium of cells exposed to hypoxia in the presence or absence of TNFα and IFNγ. Our results showed that after 24 h, hypoxia, either per se or in combination with the proinflammatory cytokines, did not induce apoptosis in MSC (Fig. 3B). Neither after 48 h, hypoxia alone did induce apoptosis in MSC. However, 48 h of cell stimulation with TNFα and IFNγ in the presence of hypoxia led to the activation of Caspases 3/7 in most of the cells (Fig. 3B and Movies S1 and S2).

To provide further evidence that the increase in Luc signal was the consequence of the hypoxia-induced Pgk promoter activation and not of MSC proliferation after in vivo transplantation, the proliferation of MSC in normoxic or hypoxic conditions was assessed in vitro. The results showed that hypoxia did not stimulate MSC proliferation in vitro when measured for up to 5 days of culture (Fig. 3C), which thus confirmed that the increase in BLI signal was a direct result of Pgk promoter activation.

Next, the activation of hypoxia following in vivo transplantation of MSC into the pancreas was further investigated by using HRE-Luc-expressing MSC and following the time-course activation of HIF-1α signalling pathways post-transplantation. The results showed a strong, yet transient, activation of hypoxia-induced signalling pathway in intrapancreatically transplanted MSC (Figs. 3D and S2A,B).

To investigate whether the reduction of BLI signal after three days from transplantation might be the consequence of locally activated apoptosis, the dynamics of BLI Luc signal from healthy and apoptotic MSC have been comparatively evaluated in vivo after transplantation into the pancreas of NOD females. To obtain apoptotic MSC, cells were pre-incubated for 48 h with TNFα and IFNγ, which resulted in ~45% apoptotic cells (Fig. S3A). The data showed the absence of the transient increase in BLI signal and a faster BLI signal disappearance of apoptotic MSC, as compared to control MSC (Fig. S3B). This data strengthened the conclusion that the local activation of MSC apoptosis might occur in vivo and this might be responsible for the rapid decline of BLI signal after MSC transplantation.

### MSC undergo apoptosis in vivo after transplantation

**Fig. 4:**
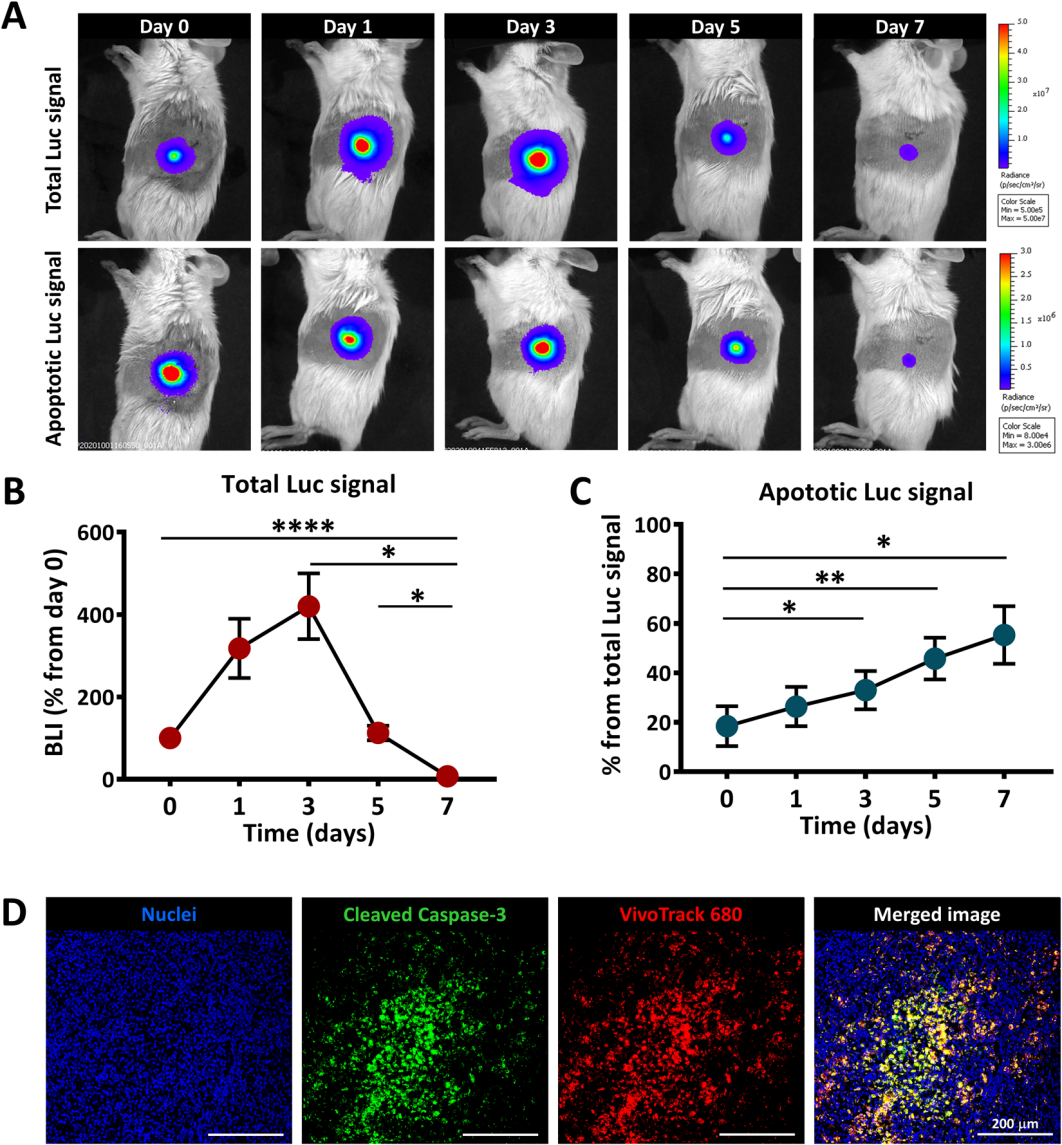
In vivo apoptosis of MSC after intrapancreatic transplantation. **A** Representative BLI time-course images obtained after injection of D-Luciferin (for detection of total viable cells) or Z-DEVD-aminoluciferin (for detection of apoptotic cells), after intrapancreatic transplantation of Luc+ MSC; **B** Quantification of BLI signal measured after D-Luciferin administration in mice; data are shown as mean ± SEM (*n* = 5); **C** Quantification of BLI signal measured after Z-DEVD-aminoluciferin administration in mice; data are shown as mean ± SEM (*n* = 5). **D** Immunofluorescence images of the pancreas of a NOD mouse at 7 days after MSC local transplantation, indicating cellular apoptosis at the site of transplantation. Note that numerous VT680-labbeled MSC were apoptotic at that time, as revealed by the presence of cells co-stained with VT680 (red pseudo-colour) and cleaved Caspase 3 (green pseudo-color).

Further analysis of the fate of grafted MSC was performed by in vivo imaging of cell apoptosis, employing a method previously documented^26,27^. In this experiment, syngeneic Luc^+^ MSC were transplanted in the pancreas and imaged after D-luciferin injection, confirming the presence of living Luc^+^ MSC. In parallel, the evaluation of Caspase 3/7 activity in Luc^+^ MSC was assessed after Z-DEVD-aminoluciferin injection (Fig. S4). Besides the transient increase in the total BLI signal noticed in the first three days after transplantation, this dual imaging technique allowed the identification of apoptosis as a key event affecting the grafted cells. Thus, the apoptotic cells appeared shortly after MSC infusion and were constantly identified at all time points thereafter at similar ratios of the total signal (Fig. 4A–C). As an apoptotic agent, the signal of Caspase 3/7 activity should be additive over time, indicating a substantial cell loss by apoptosis after syngeneic MSC transplantation in NOD mice (Fig. 4C). The in vivo caspase-mediated apoptosis assay was further confirmed on pancreatic tissues at 7 days post-transplantation, by immunohistochemistry using a specific antibody for the cleaved form of Caspase 3. The results illustrated the local presence of numerous cells co-stained with cleaved Caspase 3 and VT680 at the transplantation site (Fig. 4D).

### Syngeneic MSC transplant triggers a strong local innate immune response

Inconsistent patterns of detection of the two dyes and increased apoptotic signals after transplantation suggested that transplanted cells died and were engulfed by phagocytic cells. This hypothesis may explain the disappearance of Luc signal (marker of viable cells) concomitant with the local persistence of the fluorescent dye (that is passing to other cells within environment, which engulf MSC-derived-VT680 containing-apoptotic bodies). To explore this hypothesis, the frequency of the immune cells at the site of transplantation was evaluated in the pancreatic tissue at 7 days after intra-pancreatic transplantation. Immunofluorescence analysis showed a localized VT680 signal in the pancreas of pre-diabetic NOD females and distribution of VT680^+^ cells in the pancreatic parenchyma at distance from the transplant (Fig. S5A–B).

**Fig. 5:**
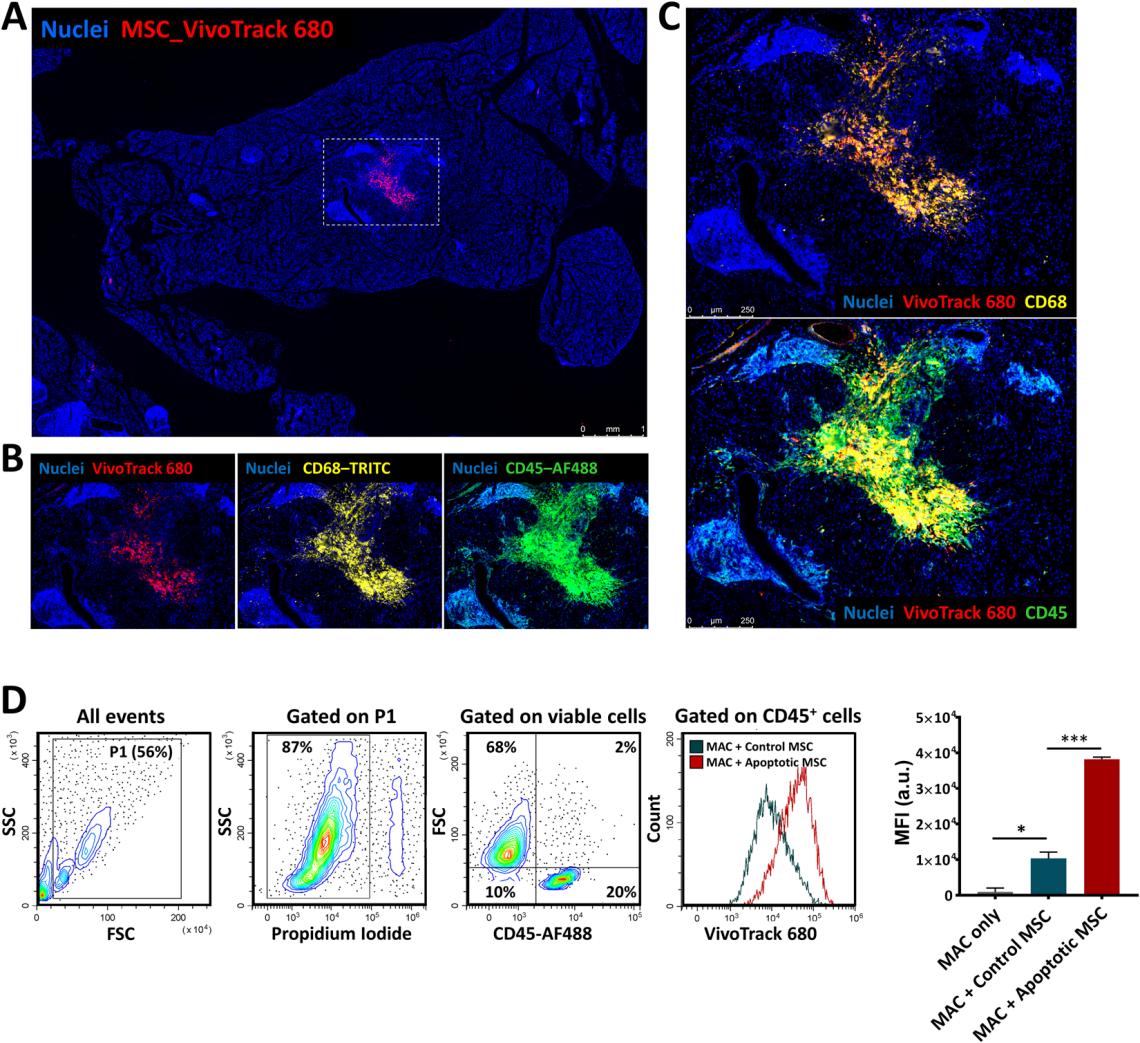
Infiltration of inflammatory cells after intrapancreatic transplantation of MSC. **A** Low-magnification scan of the pancreas at 7 days after transplantation of Luc^+^ VT680^+^ MSC; box indicates the area with a high VT680 signal; **B** Immunofluorescence images of the region indicated in (**A**). CD68 immunostaining denotes macrophages infiltrated at the transplantation site; CD45 immunostaining demarcates hematopoietic cells; **C** High-resolution microscopy of the region indicated in (**A**) showing the presence of cells with co-localized signal of VT680 and CD68 molecule (upper image) and co-localized signal of VT680 and CD45 molecule (lower image), thus suggesting the presence of macrophages that engulfed VT680^+^ MSC or VT680^+^ MSC-derived apoptotic bodies. **D** Flow cytometry analysis of peritoneal macrophages isolated from syngeneic mice co-cultured with healthy (control) or apoptotic MSC. Apoptotic MSC were obtained after 48-h culture with pro-inflammatory cytokines. Note the increased phagocytic activity of macrophages when in presence of apoptotic cells as compared to healthy cells.

We were particularly interested in estimating the distribution of MSC into the pancreatic islets, which are known to display variable degrees of inflammation in NOD mice. At 7 days post-transplantation, histological analysis showed a reduced localization of VT680^+^ cells within the islets and only incidental presence of the cells at islet borders, thus indicating a weak capacity of MSC to migrate to sites of inflammation in this murine model of diabetes. Moreover, VT680 signals overlayed to CD45 positive cells, which have been identified as monocyte-macrophage cells by positive CD68 staining (Fig. 5A–C). Additional macrophages were also seen in the vicinity of MSC (Fig. 5B, C), indicating that inoculation of large numbers of MSC was accompanied by a robust recruitment of immune cells to the site of implantation. To further confirm the clearance of apoptotic MSC by macrophages, in vitro co-culture experiments of peritoneal macrophages with control or apoptotic Luc^+^ VT680^+^ MSC were performed. Peritoneal macrophages were isolated using an immunomagnetic negative selection protocol and confirmed as being CD45^+^ CD11b^+^ F4/80^+^ cells, with more than 93% purity and 95% viability (Fig. S6A–B). After one hour of co-incubation, the not-attached cells were removed and the attached cells were pooled and analysed by flow cytometry. The results showed that peritoneal macrophages had increased phagocytic activity in the presence of apoptotic MSC, as compared to control MSC (Fig. 5D). This in vitro data put forth an argument that favours our hypothesis, thus further suggesting that the persistence of the fluorescent signal in vivo is, at least in part, due to the host macrophages that are accumulated at the site of transplantation and take up the apoptotic bodies and dead cells.

In summary, the intrapancreatic transplantation of MSC in NOD mice resulted in the activation of hypoxia, and subsequently of apoptosis signalling in the transplanted cells, which produced a strong immune cell response at the transplantation site, followed by the disappearance of MSC from the targeted organ.

## Discussion

MSC are under extensive research as tools for cell-based therapy, yet important mechanistic aspects are still incompletely understood^3^. Our study assessed migration and survival of syngeneic Luc^+^ MSC administered by different routes in the NOD mouse model of inflammatory insulitis. The novel findings of our study are as follows: (i) the survival of syngeneic MSC after transplantation in the NOD prediabetic mouse is limited to about 7 days, irrespective on the transplantation route; (ii) intrapancreatically transplanted MSC transiently activated hypoxia-induced signalling pathway, which peaked between 1 and 3 days after transplantation; (iii) in the presence of a proinflammatory environment, hypoxia-activated Caspase-3/7-mediated apoptosis, which gradually affected the transplanted cells; (iv) MSC-derived apoptotic bodies are engulfed by locally accumulated macrophages, within one week after transplantation; (v) the methods of labelling the cells for further in vivo monitorization must be regarded with cautions, as they are subjected to limitations, which may include either false-positive results regarding the local persistence of the transplanted cells, or false-positive data regarding the local proliferation of the transplanted cells.

Allogeneic MSC may be preferred over syngeneic MSC for the treatment of autoimmune diseases, given the decreased overall effectiveness of the host-derived cells which may have exclusively intrinsic defects in their therapeutic properties^28,29^. Still, to better understand the biology of MSC after transplantation, syngeneic MSC were used in this study, which circumvented the risk of the immunogenic response.

Although the intravenous route is the most popular in cell therapy, and the transmigration of intravascularly infused MSC through the endothelial barrier is a well-described mechanism^30^, the number of cells detected after transplantation is much lower than the administered number of cells^4,31^. Moreover, while histological techniques are still used as the gold standard to assess the migration or survival of the grafted cells, false-positive results are common when signals emanate from dead cells^32^.

When intravenously infused, MSC did not migrate to the pancreas, being rather entrapped into the lungs. Therefore, this delivery route was concluded as ineffective for directing MSC to the pancreas and associated lymphoid organs. In our model, MSC persistence within the lungs after intra-venous administration exceeded the previously reported lifetime^6,33,34^, with signals detected even at 7 days after transplantation.

In contrast to intravenous administration, we report here that direct delivery of MSC into the pancreas was associated with a minor dissipation of the cells within the organ. However, MSC delivery into the spleen was transiently associated with partial migration of the cells to the liver, which can emphasize the functional relationship between these two organs in the so-called hepato-splenic regulatory axis, previously reported as an intersection linking immunity, pathogen clearance and metabolism in various conditions^35^.

Previous data showed that transplanted Luc^+^ MSC survived much longer in fully immunocompetent syngeneic versus allogeneic recipients^36^, while cells expressing immunogenic proteins were invariably recognized and rejected in nontolerant immunocompetent recipients^37,38^. Recent studies demonstrated that intraarticularly injected alkaline phosphatase-expressing MSC were rapidly identified, rejected and cleared in wild-type syngeneic recipients^39^.

Using a dual MSC tracking system composed of transgenic expression of Luc gene and the VT680 fluorescent dye, we detected a transient increase of BLI signal soon after implantation, which generally persisted for 3 days. We demonstrate that the increased BLI signal was at least partially caused by activation of the Pgk promoter in vivo under hypoxic conditions. The Pgk promoter used in our study was previously reported to have relative advantages over the CMV promoter, including more stable and long-term protein expression^40,41^. However, there was evidence that promoter silencing occurred in vivo and led to loss of transgene expression^42^.

Soon after a transient increase in BLI signal, the Luc activity rapidly decreased, irrespective of the transplantation route, likely as a result of cell death. This reporter protein was suggested to be safe, with no induction of the immune rejection of Luc^+^ MSC^36,43^. Besides, the third-generation lentiviral-based system used in this study is reportedly widely accepted and has been recently used in multiple clinical trials^44,45^. However, based on previous reports on the immunogenic sensitization elicited by the reporter genes in immunocompetent animals^37,38,46^, we cannot exclude that the reporter protein attracted immune recognition and rejection. On the other hand, the experimental data may also be influenced by the recipient strain and the donor cell source (allogeneic versus syngeneic transplants)^36,37^.

Our data emphasizing in vivo apoptosis and activation of immune responses after MSC transplantation should be considered in reference to the immunomodulatory activity and the ability of allogeneic MSC to defend themselves from rejection (the alleged immune privilege). Allogeneic MSC are indeed rejected after infusion^10,47^, however, apoptotic MSC still retains the immunomodulatory activity^9–12,47^, which could be the main mechanism of action and may explain the great difficulty in tracking these cells in vivo. Moreover, a critical look must be taken on our in vivo apoptosis imaging data as non-apoptotic caspase-3 enzymatic activity has been observed in MSC or hematopoietic stem cells in other reports^48,49^.

In conclusion, this study provides details regarding the fate of exogenously administered MSC in mice with autoimmune insulitis. We demonstrate that transplanted MSC had a short lifespan and a limited dynamic distribution to other sites. Moreover, soon after infusion, syngeneic MSC underwent caspase-mediated apoptosis and elicited a strong activation of the innate immune system. Our study argues for a “hit and die” mechanism of action but further investigations are required to shed light on the molecular crosstalk between inoculated MSC and host-immune cells.

## Materials and methods

### Animals

NOD/ShiLtJ (NOD; Stock No: 001976) mice were purchased from the Jackson Laboratory and bred in the local animal facility. Mice were maintained under specific pathogen-free conditions in a controlled environment with a 12/12-h light/dark cycle, 21 °C and 55–60% humidity, and had access to chow and water ad libitum.

### MSC isolation and labelling

The cells were isolated from the bone marrow of 6 to 8-week-old male NOD mice using the method previously described^22^. Cell characterization confirmed the presence of MSC specific markers (Sca-1, CD105, and CD44), the absence of hematopoietic markers (CD45 and CD11b) and the trilineage differentiation potential into osteogenic, adipogenic and chondrogenic lineages, as previously reported^50–52^. Cells were used between the 7th and 10th passages. To track the cells in vivo after transplantation, cells were labelled by transduction with a third-generation lentiviral system to stably express Luc transgene^50^. Briefly, the lentiviral particles were assembled by using the packaging plasmids pRSV-Rev, pMDLg/pRRE, pMD2.G (gifts from Didier Trono, Addgene plasmids # 12253, # 12251, and # 12259)^53^, and the transfer plasmid for Luc (a gift from Eric Campeau, Addgene plasmid # 21471)^40^. Transduced cells preserved their properties, being able to differentiate into osteocytes, adipocytes and chondrocytes, when cultured under appropriate conditions, as previously reported^50^. In some experiments, Luc+ cells were additionally labelled with VivoTrack 680 (VT680) just prior to transplantation. VT680 labelling of MSC was performed according to the manufacturer’s recommendations (PerkinElmer, NEV12000). Briefly, cell suspension (10^6^ cells/ml) in phosphate-buffered saline (PBS) was incubated with 50 μg/ml VT680 for 15 min, at room temperature, in the dark, and washed three times with PBS containing 1% FBS before use.

### MSC transplantation

Double-labelled MSC (that expressed Luc and VT680) were transplanted by different routes, i.e., intravenous, intrapancreatic, intrasplenic and subcutaneous, in pre-diabetic 12-week-old NOD females. Twenty-four hours before transplant, labelled MSC were plated at 20,000 cells/cm^2^ so that to form an 80% confluent culture of actively dividing cells on the transplant day. Mice were anaesthetized with a mixture of ketamine-xylazine-acepromazine (80-10-2 mg/kg bodyweight) and cell suspension prepared by trypsinization (using 0.25% Trypsin-EDTA, ThermoFisher) was resuspended in ice-cold PBS, pH 7.4, and kept on ice until injection. For subcutaneous transplantation, aliquots of 5 × 10^5^ cells in 50 μl PBS/site were injected in both the interscapular and inguinal regions. For intravenous transplantation, a same number of cells was resuspended in 250 μl PBS and slowly injected (in the course of 10–15 min per animal) in the lateral tail vein. For intrasplenic and intrapancreatic transplantation, small laparotomies were performed under sterile conditions to expose the tail of the spleen and the pancreas, respectively. A number of 5 × 10^5^ cells (resuspended in 50 or 100 μl PBS for intra-pancreatic or intrasplenic transplantation, respectively) were transplanted using a 1700 Series Hamilton syringe with a 28 G needle. After transplant, the incisions were sutured with a 6/0 Optilene polypropylene monofilament (BBraun) and Baneocin® 250 UI/5000 UI antibiotic ointment was applied on the surgical suture. Complete healing was observed in all animals, with complete epithelization by 7 days post-transplant. For analgesia, intrasplenic and intrapancreatic transplanted animals were subcutaneously injected with buprenorphine hydrochloride (0.1 mg/kg, Temgesic) after laparotomy.

### In vivo imaging

The survival and migration of MSC was monitored up to 7 days post- transplant, using an IVIS Spectrum in vivo imaging system (PerkinElmer). For FLI, mice were anesthetized with 1.5% isoflurane (Isoflutek, 710004), placed in the IVIS imaging box and imaged dorsally or laterally. For BLI, anesthetized mice were injected intraperitoneally with D-Luciferin Potassium Salt (PerkinElmer, 122799) (150 mg/kg bodyweight) and 12 min later, the total signal produced by transplanted cells was imaged. In experiments for in vivo apoptosis imaging, a second injection with Z-DEVD-aminoluciferin (VivoGlo Caspase 3/7 Substrate, Promega, P1781) 100 mg/kg bodyweight was applied at 4–6 h after D-luciferin injection, at a time when BLI signal produced by D-luciferin was totally extinct. Fluorescence and bioluminescence images of live animals or isolated organs were analysed using Living Image 4.5 software (PerkinElmer) by manually defining the regions of interest. Imaging data were normalized and expressed as radiance (p/s/cm²/sr) for bioluminescence or Radiant Efficiency ([p/s/cm²/sr]/[µW/cm²]) for fluorescence, and the colour scale was adjusted according to the strength of signal. To quantify the fluorescent signal, spectral unmixing analysis was performed to extract the autofluorescence from the specific signal. For quantification, the relative signal intensity was calculated as a percentage from the signal intensity at day 0 (30 min post-transplantation).

In vivo hypoxia visualization was performed as previously described^22^. Briefly, 24 h prior to transplantation, MSC were transiently transfected with HRE-luciferase plasmid (Addgene # 26731, a gift from Navdeep Chandel)^54^ by electroporation (NEPA21; Nepagene). This Luciferase reporter construct contains tree hypoxia response elements (HRE) from the Pgk-1 gene upstream of firefly luciferase. For hypoxia visualization in vitro, HRE-luc transfected MSC were seeded at pre-confluence (20,000 cells/cm^2^) and, 24 h later, the cells were incubated in 2% O_2_ atmosphere (hypoxia) for another 24 h. Hypoxic condition was achieved using a dedicated hypoxia station (Whitley H35 Hypoxystation, Don Whitley Scientific Limited, U.K.). As control, transfected cells were maintained in 21% O_2_ (normoxia). BLI signal was measured with IVIS Spectrum by imaging the cells immediately after the addition of 150 μg/ml D-Luciferin. To mimic the proinflammatory environment in vitro, MSC were stimulated with TNFα (R&D Systems, 410-MT) and IFNγ (R&D Systems, 485-MI) at 20 ng/ml each, under either normoxia or hypoxia for 24 h.

### Cell proliferation assay

MSC were plated in two six-well cell culture plates (Eppendorf, 0030720113) at a density of 50,000 cells/well and grown for 5 days in either normoxia or hypoxia. Cell proliferation was determined after 1, 3, and 5 by counting the cells (after trypsinization). The experiment was performed three times with biological duplicates for each time point.

### Apoptosis assays

The apoptosis assays were employed to estimate the effect of double labelling of MSC with Luc and VT680, as well as to quantify the cell death induced in MSC after the treatment with TNFa and IFNγ (20 ng/ml, each) for 48 h. To estimate the effect of double labelling, 10^5^ MSC were incubated with 5 µl APC Annexin V (Biolegend, 640920) and 5 μg/ml Propidium Iodide (Sigma-Aldrich, P4170) in 100 μl, for 15 min in the dark. To determine apoptosis of MSC in pro-inflammatory conditions, 10^5^ cells (collected as both floating and attached cells) were stained with CellEvent™ Caspase-3/7 Green ReadyProbes™ Reagent (ThermoFisher Scientific, R37111), according to the manufacturer’s instructions. Cells stained with Annexin V and PI or CellEvent™ Caspase-3/7 were analysed by flow-cytometry. At least 100,000 events were recorded for each sample, using a CytoFLEX Flow Cytometer (Beckman Coulter, U.S.A.) and the acquired data were analysed using CytExpert version 2.1 software.

Furthermore, apoptosis induced by hypoxia, in the presence and absence of the pro-inflammatory cytokines was also evidenced by time-lapse fluorescence microscopy using a PAULA Smart Cell Imager (Leica Microsystems, Germany). After 24 h of treatment, CellEvent™ Caspase-3/7 reagent was added onto the cells and the cells were imaged for the next 24 h at a time interval of 20 min.

### In vitro phagocytosis assay

Peritoneal macrophages were obtained from 8 to 12-week-old male NOD mice by lavage with 5 ml of ice-cold PBS, followed by macrophage purification using EasySep™ Mouse Monocyte Isolation Kit (Stem Cells Technologies, #19861). The purified cells were cultured in four-well plate wells at 2.5 × 10^5^ cells/well for 24 h in RPMI medium supplemented with 10%FBS before being washed and further incubated with VT680-labelled MSC (treated or not-treated in inflammatory conditions) at a cell ratio of 1:5 (macrophages/MSC). After one hour of co-incubation, the not-adhered MSC were removed by three washes with PBS, and the adhered cells (MSC and macrophages) were collected with trypsin. A cell suspension containing 10^5^ cells was then incubated in 100 µl FACS buffer with FITC-labelled anti-CD45 antibody and the presence of VT680 within macrophages was analysed by flow cytometry to estimate the extent to which peritoneal macrophages engulfed normal or apoptotic MSC.

### Histology and image acquisition

The pancreas was isolated and processed by overnight fixation into PBS with 1.5% paraformaldehyde and 0.1% glutaraldehyde, followed by 24-h cryoprotection in PBS with 30% sucrose. Fixed samples were soaked in OCT (Optimal Cutting Temperature) compound and frozen on a metal block immersed in liquid nitrogen, before being sliced in seven-μm cryosections. For apoptosis assay, sections were incubated with anti-Cleaved Caspase-3 (Asp175) antibody (Cell Signaling Technology, #9661) for 2 h at room temperature followed by incubation with NL557-conjugated Anti-Rabbit IgG secondary antibody (R&D Systems, NL004), for one hour in the dark. For macrophage identification, the slides were incubated with anti-CD68 monoclonal antibody (BioLegend, 137001) overnight at 4 °C, followed by incubation with AF488-labelled anti-CD45 monoclonal antibody (BioLegend, 103122), 2 h at room temperature and dark and then by incubation with TRITC-conjugated Anti-Rat IgG secondary antibody (Sigma-Aldrich, T5778) for one hour in the dark. Two-three sections at 150 μm apart were imaged per each animal. Image acquisition was performed using a Leica DMi8 inverted fluorescent microscope equipped with HC PL APO 10x/0.45 NA dry, and HC PL APO 40x/1.3 NA oil objectives. Fluorophores were excited with a multi-LED Spectra-X light source (Lumencor) and images were captured with a sCMOS camera Leica DFC9000 and subsequently processed with Leica LAS X software. Tile scan z-stack images were acquired with LAS X Navigator module and mosaic merged with smooth overlap blending and then digitally processed for extended depth of field.

### Statistical analysis

Data were expressed as mean ± SEM and analysed with GraphPad Prism 7.0. Longitudinal comparisons between different groups were performed with one-way or two-way analysis of variance (ANOVA) with Tukey’s post-test analysis. Statistical significance was defined as *p* < 0.05.

## Supplementary information

NOD-MSC in hypoxia

NOD-MSC in hypoxia _ TNFa+IFNg

Supplemental material
