# Supplementary material for: Short lifespan of syngeneic transplanted MSC is a consequence of in vivo apoptosis and immune cell recruitment in mice
Source: Cell Death Dis. 2021 Jun 2;12(6):566. doi: 10.1038/s41419-021-03839-w (PMC8169682; doi:10.1038/s41419-021-03839-w)
Supplement: Supplementary file 3 — Supplemental material [file 41419_2021_3839_MOESM3_ESM.pdf]

Supplemental Information

Short lifespan of syngeneic transplanted MSC is a consequence of *in vivo* apoptosis and immune cell recruitment in mice

Mihai Bogdan Preda[1]*, Carmen Alexandra Neculachi[1], Ioana Madalina Fenyo[2], Ana-Maria Vacaru[2], Mihai Alin Publik[1], Maya Simionescu[1,2], Alexandrina Burlacu[1]

[1]Laboratory of Stem Cell Biology, Institute of Cellular Biology and Pathology "Nicolae Simionescu", Bucharest, Romania

[2]Laboratory of Gene Regulation and Molecular Therapies, Institute of Cellular Biology and Pathology "Nicolae Simionescu", Bucharest, Romania

Supplemental Figures

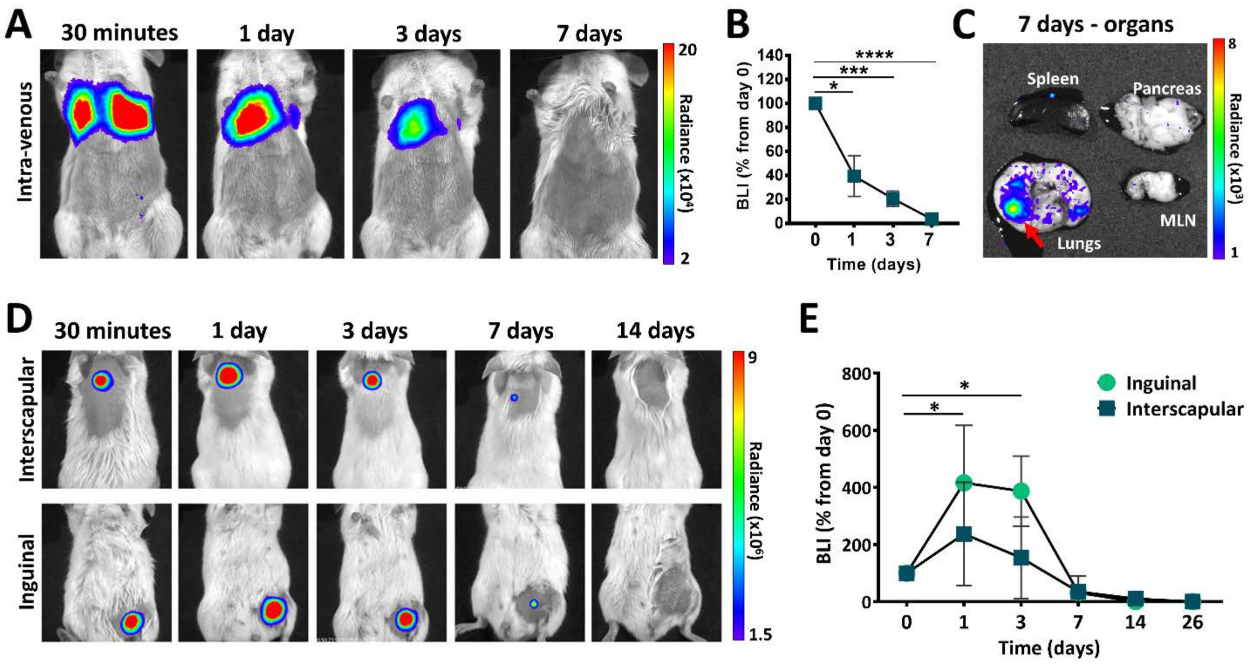

Supplemental Figure 1. Biodistribution and survival of MSC after intravenous (A-C) or subcutaneous (D-E) transplantation in pre-diabetic NOD females. (A) BLI time-course images of a mouse transplanted intravenously with Luc^+ MSC; (B) Quantitative data measured from (A) are shown as mean ± SEM (n=5) (*$p < 0.05$, ***$p<0.0005$, ****$p<0.0001$, one-way ANOVA followed by Tukey's test). (C) BLI image of isolated organs at 7 days after intravenous transplantation of Luc^+ MSC. Red arrow indicates the BLI signal localized in the lungs; (D) Representative images of BLI signal of Luc+ MSC after subcutaneous transplantation in two distinct anatomical regions in one pre-diabetic NOD female; (E) Quantitative data measured from (D) as mean values ± SEM (n=8) (*$p < 0.05$, two-way ANOVA followed by Tukey's test).

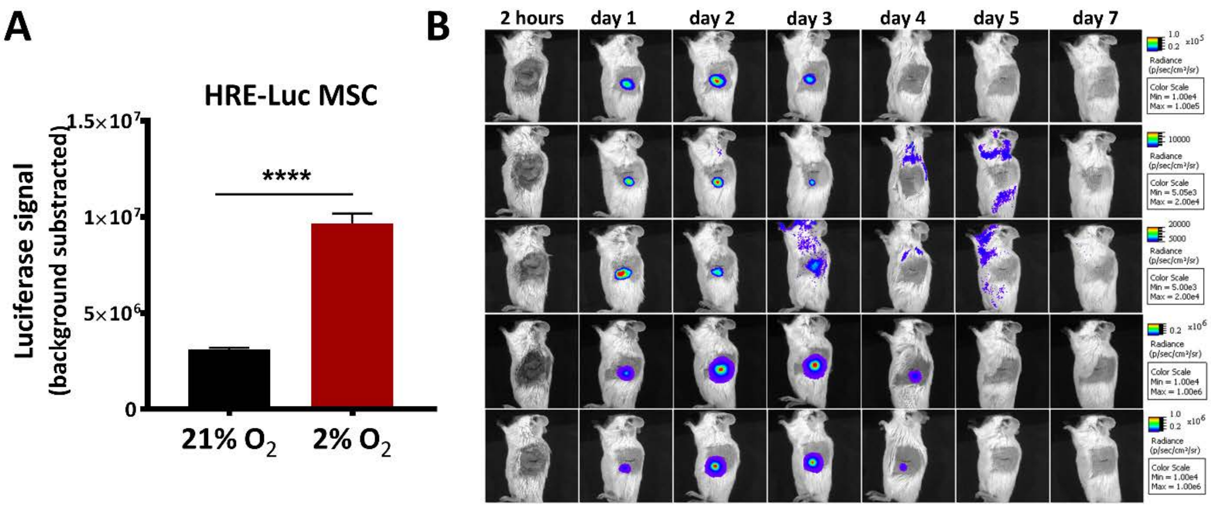

Supplemental Figure 2. Time-course activation of hypoxia in MSC after intra-pancreatic transplantation in pre-diabetic NOD females. (A) Assessment of transfection efficiency with the HRE-luciferase plasmid in MSC by analysis of Luc signal in transfected MSC after 24-hour culture under hypoxic conditions (2% O_2). The data represent mean+/- SEM of three independent experiments. (***p < 0.005, Student's t test). (B) Time-course evaluation of 5 different mice after intra-pancreatic transplantation of HRE-Luc-expressing MSC. Note the activation of hypoxia signaling starting with day 1 after transplantation. The quantitative data is given in the main manuscript.

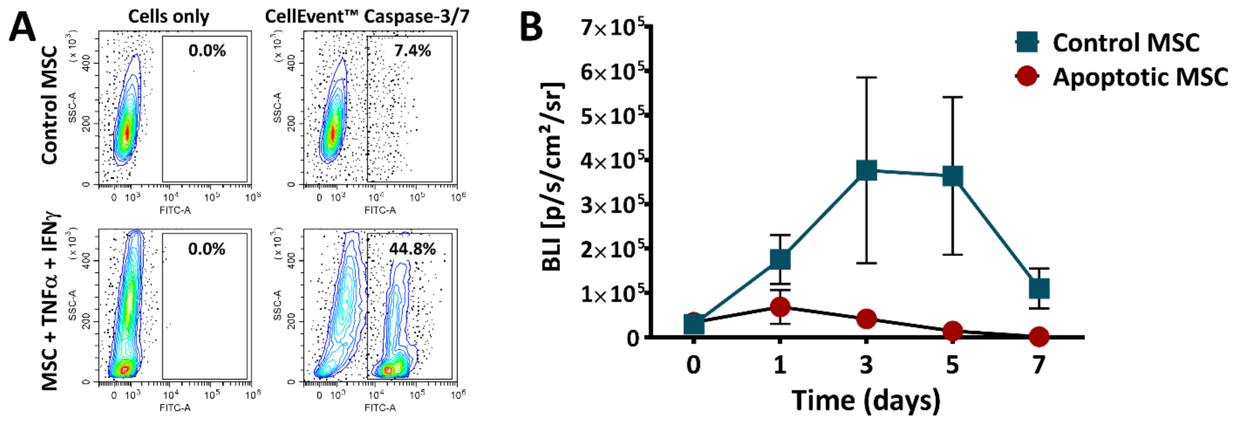

Supplemental Figure 3. Evaluation of the signal produced by transplantation of apoptotic MSC as a direct comparison to the transplantation of healthy MSC. (A) Apoptotic MSC were obtained by 48-hour treatment of cells with a mixture of 20 ng/ml TNFα and 20 ng/ml IFNγ. Note that around half of MSC were positive for the active form of Caspase-3/7 after the treatment. (B) The survival of control and apoptotic MSC (as whole populations of cultured cells, not-treated and treated with TNFα/IFNγ, respectively) after intra-pancreatic transplantation in pre-diabetic NOD females. Data are shown as mean ± SEM (n=5 animals per each group).

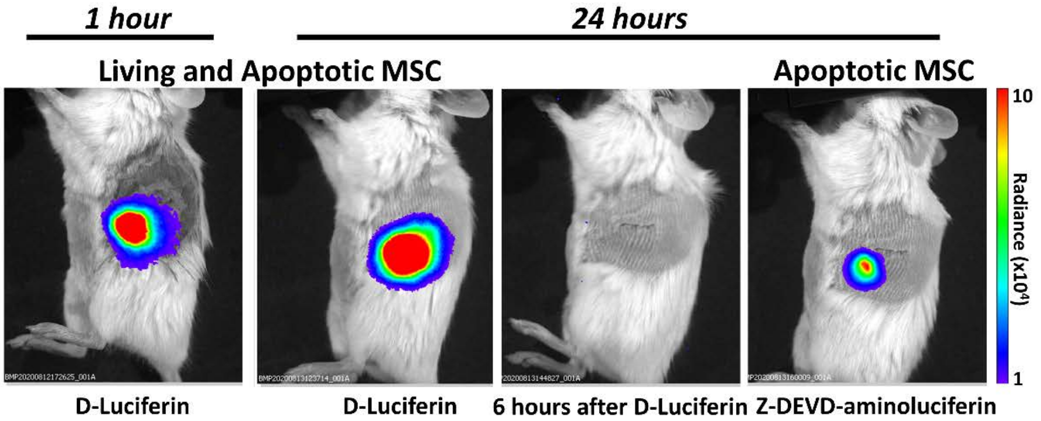

Supplemental Figure 4. In vivo apoptosis of MSC after intrapancreatic transplantation. BLI images of a prediabetic NOD female injected intrapancreatically with Luc^+ MSC. Luc signal was detected after D-Luciferin administration (total signal of both healthy and apoptotic cells) or Z-DEVD-aminoluciferin administration (that detected only the apoptotic cells).

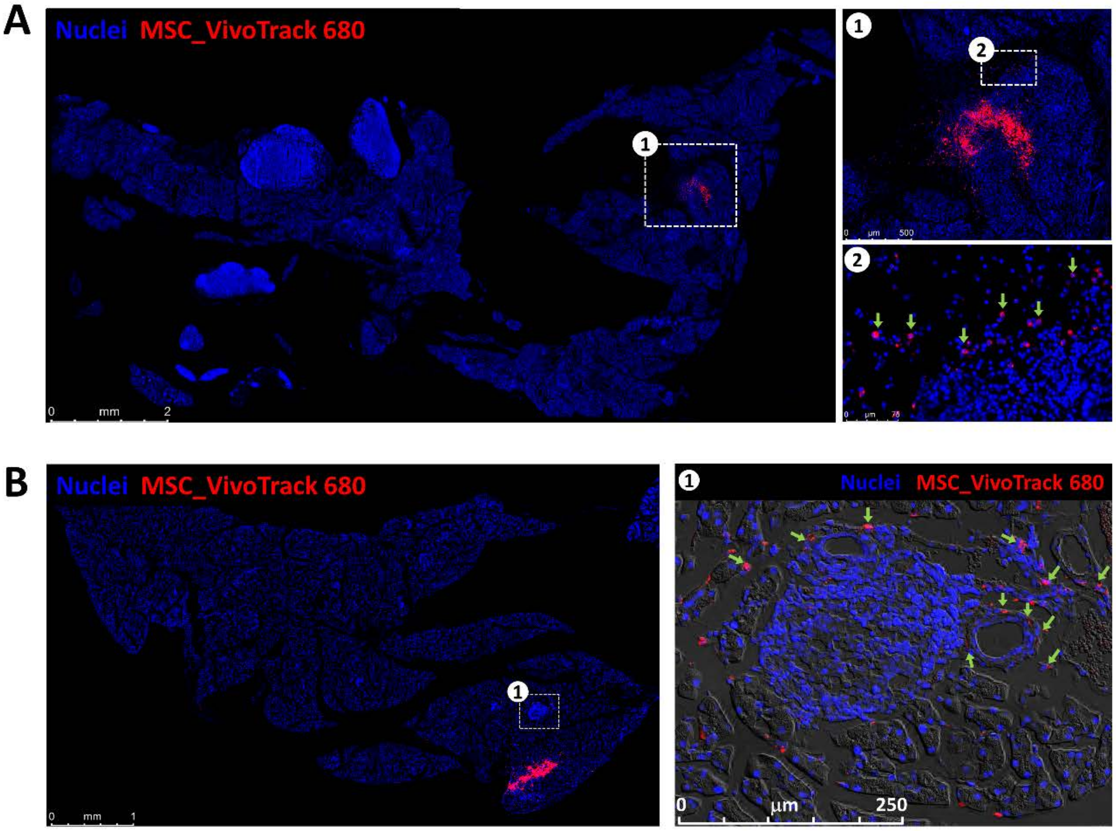

Supplemental Figure 5. Transplanted MSC do not infiltrate pancreatic islets in NOD mice. (A) Immunofluorescence image at low magnification obtained from of a section of the pancreas stained with Hoechst 33258 (for cell nuclei) at 7 days after intra-pancreatic transplantation of MSC labelled with Luc and VT680. A higher magnification of the box 1 indicating the transplantation site is showed in the images on the right. Note in the Box 2 the existence of VT680-expressing cells with round morphology (arrows); (B) Immunofluorescence image of a pancreas section showing the transplantation site (the intense red signal) and a nearby large pancreatic islet (box 1). At higher magnification, the box 1 shows VT680-labelled cells surrounding the islet and being located around the vessels, but without penetrating the islet.

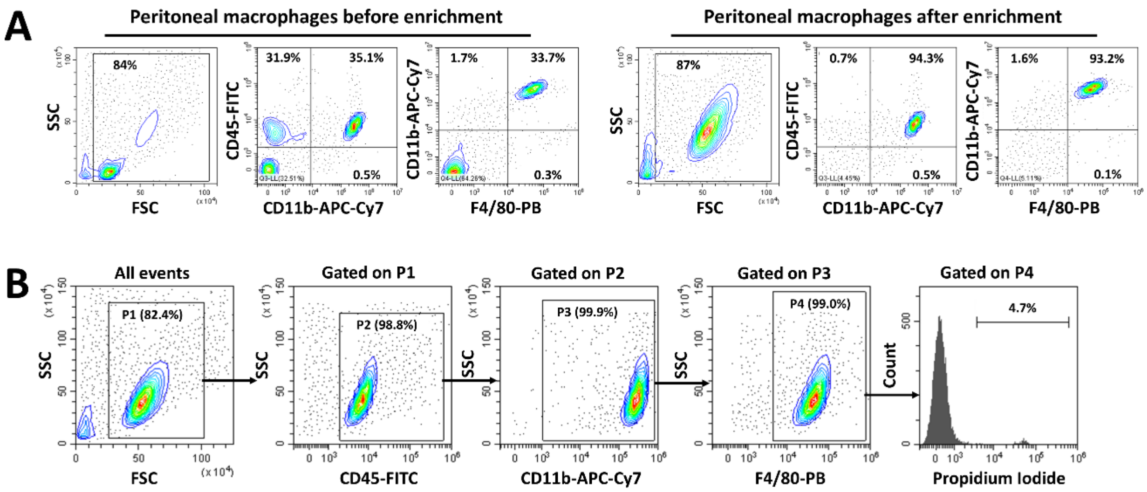

Supplemental Figure 6. (A) Frequencies of macrophages ($CD45^+/CD11b^+/F4/80^+$) in unseparated peritoneal lavage (left) versus purified cells, obtained by enrichment of macrophages with EasySep™ Mouse Monocyte Isolation Kit (right). Note the high purity of peritoneal macrophages in the cell suspension after enrichment. (B) The viability of purified peritoneal macrophages after enrichment, as determined by Propidium iodide staining. Note the high percentage of viable cells after purification.
